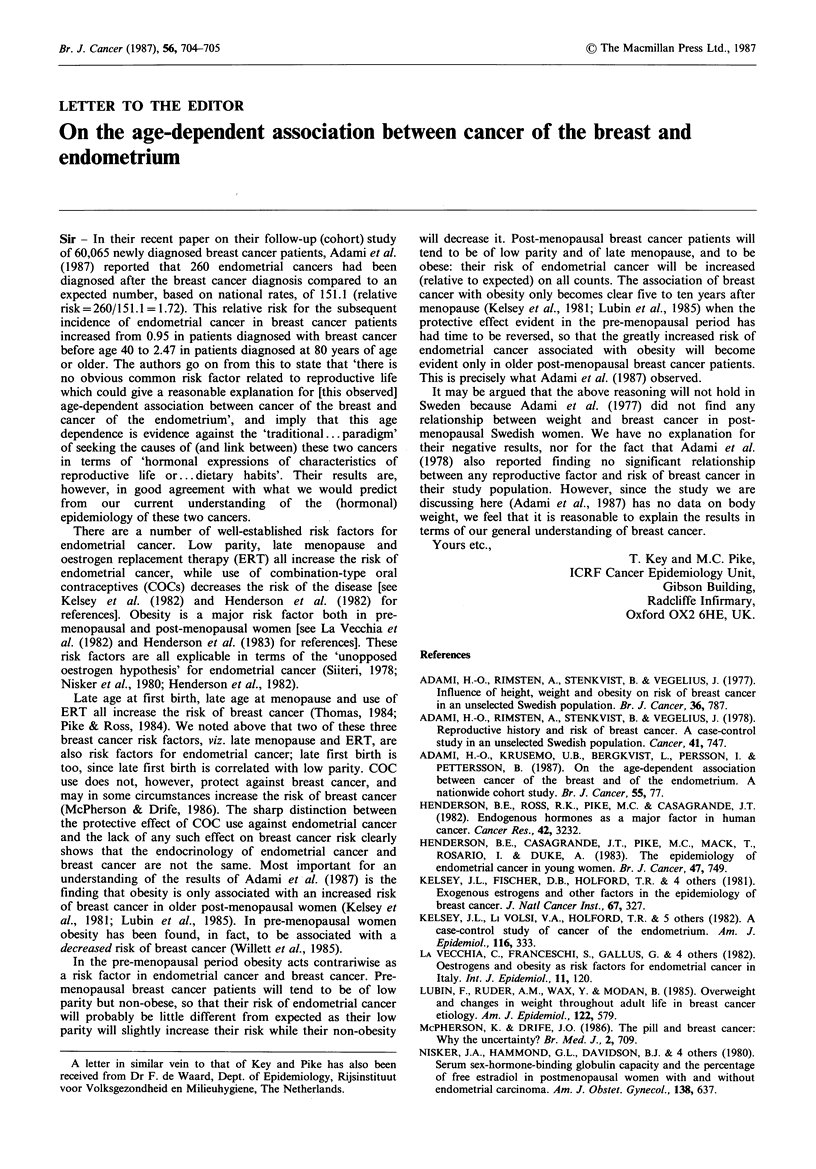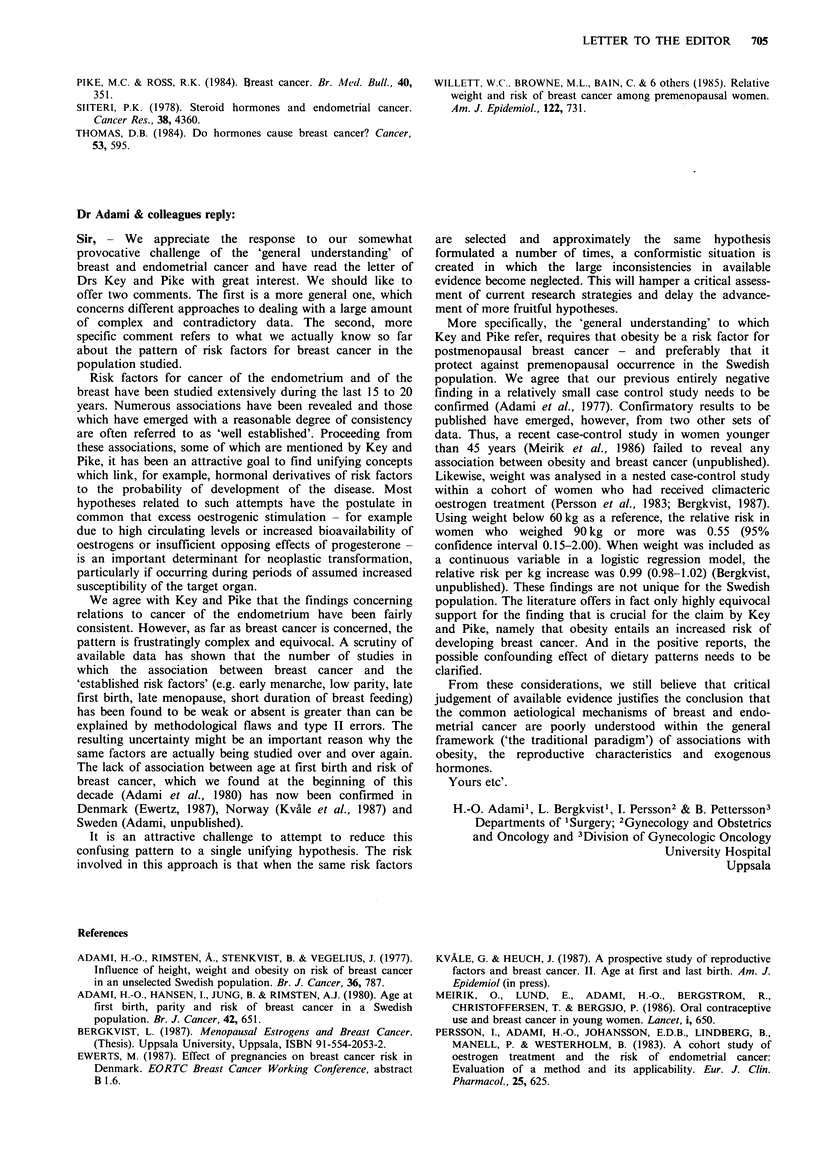# On the age-dependent association between cancer of the breast and endometrium.

**DOI:** 10.1038/bjc.1987.270

**Published:** 1987-11

**Authors:** T. Key, M. C. Pike


*Br. J. Cancer* (1987), **56**, 704–705 © The Macmillan Press Ltd., 1987

LETTER TO THE EDITOR

### On the age-dependent association between cancer of the breast and endometrium

**Sir** – In their recent paper on their follow-up (cohort) study of 60,065 newly diagnosed breast cancer patients, Adami *et al.* (1987) reported that 260 endometrial cancers had been diagnosed after the breast cancer diagnosis compared to an expected number, based on national rates, of 151.1 (relative risk = 260/151.1 = 1.72). This relative risk for the subsequent incidence of endometrial cancer in breast cancer patients increased from 0.95 in patients diagnosed with breast cancer before age 40 to 2.47 in patients diagnosed at 80 years of age or older. The authors go on from this to state that 'there is no obvious common risk factor related to reproductive life which could give a reasonable explanation for [this observed] age-dependent association between cancer of the breast and cancer of the endometrium', and imply that this age dependence is evidence against the 'traditional... paradigm' of seeking the causes of (and link between) these two cancers in terms of 'hormonal expressions of characteristics of reproductive life or... dietary habits'. Their results are, however, in good agreement with what we would predict from our current understanding of the (hormonal) epidemiology of these two cancers.

There are a number of well-established risk factors for endometrial cancer. Low parity, late menopause and oestrogen replacement therapy (ERT) all increase the risk of endometrial cancer, while use of combination-type oral contraceptives (COCs) decreases the risk of the disease [see Kelsey *et al.* (1982) and Henderson *et al.* (1982) for references]. Obesity is a major risk factor both in pre-menopausal and post-menopausal women [see La Vecchia *et al.* (1982) and Henderson *et al.* (1983) for references]. These risk factors are all explicable in terms of the 'unopposed oestrogen hypothesis' for endometrial cancer (Siiteri, 1978; Nisker *et al.*, 1980; Henderson *et al.*, 1982).

Late age at first birth, late age at menopause and use of ERT all increase the risk of breast cancer (Thomas, 1984; Pike & Ross, 1984). We noted above that two of these three breast cancer risk factors, *viz.* late menopause and ERT, are also risk factors for endometrial cancer; late first birth is too, since late first birth is correlated with low parity. COC use does not, however, protect against breast cancer, and may in some circumstances increase the risk of breast cancer (McPherson & Drife, 1986). The sharp distinction between the protective effect of COC use against endometrial cancer and the lack of any such effect on breast cancer risk clearly shows that the endocrinology of endometrial cancer and breast cancer are not the same. Most important for an understanding of the results of Adami *et al.* (1987) is the finding that obesity is only associated with an increased risk of breast cancer in older post-menopausal women (Kelsey *et al.*, 1981; Lubin *et al.*, 1985). In pre-menopausal women obesity has been found, in fact, to be associated with a *decreased* risk of breast cancer (Willett *et al.*, 1985).

In the pre-menopausal period obesity acts contrariwise as a risk factor in endometrial cancer and breast cancer. Pre-menopausal breast cancer patients will tend to be of low parity but non-obese, so that their risk of endometrial cancer will probably be little different from expected as their low parity will slightly increase their risk while their non-obesity will decrease it. Post-menopausal breast cancer patients will tend to be of low parity and of late menopause, and to be obese: their risk of endometrial cancer will be increased (relative to expected) on all counts. The association of breast cancer with obesity only becomes clear five to ten years after menopause (Kelsey *et al.*, 1981; Lubin *et al.*, 1985) when the protective effect evident in the pre-menopausal period has had time to be reversed, so that the greatly increased risk of endometrial cancer associated with obesity will become evident only in older post-menopausal breast cancer patients. This is precisely what Adami *et al.* (1987) observed.

It may be argued that the above reasoning will not hold in Sweden because Adami *et al.* (1977) did not find any relationship between weight and breast cancer in post-menopausal Swedish women. We have no explanation for their negative results, nor for the fact that Adami *et al.* (1978) also reported finding no significant relationship between any reproductive factor and risk of breast cancer in their study population. However, since the study we are discussing here (Adami *et al.*, 1987) has no data on body weight, we feel that it is reasonable to explain the results in terms of our general understanding of breast cancer.

Yours etc.,

T. Key and M.C. Pike,
ICRF Cancer Epidemiology Unit,
Gibson Building,
Radcliffe Infirmary,
Oxford OX2 6HE, UK.

A letter in similar vein to that of Key and Pike has also been received from Dr F. de Waard, Dept. of Epidemiology, Rijsinstituut voor Volksgezondheid en Milieuhygiene, The Netherlands.

### References

ADAMI, H.-O., RIMSTEN, A., STENKVIST, B. & VEGELIUS, J. (1977). Influence of height, weight and obesity on risk of breast cancer in an unselected Swedish population. *Br. J. Cancer,* **36,** 787.

ADAMI, H.-O., RIMSTEN, A., STENKVIST, B. & VEGELIUS, J. (1978). Reproductive history and risk of breast cancer. A case-control study in an unselected Swedish population. *Cancer,* **41,** 747.

ADAMI, H.-O., KRUSEMO, U.B., BERGKVIST, L., PERSSON, I. & PETTERSSON, B. (1987). On the age-dependent association between cancer of the breast and of the endometrium. A nationwide cohort study. *Br. J. Cancer,* **55,** 77.

HENDERSON, B.E., ROSS, R.K., PIKE, M.C. & CASAGRANDE, J.T. (1982). Endogenous hormones as a major factor in human cancer. *Cancer Res.,* **42,** 3232.

HENDERSON, B.E., CASAGRANDE, J.T., PIKE, M.C., MACK, T., ROSARIO, I. & DUKE, A. (1983). The epidemiology of endometrial cancer in young women. *Br. J. Cancer,* **47,** 749.

KELSEY, J.L., FISCHER, D.B., HOLFORD, T.R. & 4 others (1981). Exogenous estrogens and other factors in the epidemiology of breast cancer. *J. Natl Cancer Inst.,* **67,** 327.

KELSEY, J.L., LI VOLSI, V.A., HOLFORD, T.R. & 5 others (1982). A case-control study of cancer of the endometrium. *Am. J. Epidemiol.,* **116,** 333.

LA VECCHIA, C., FRANCESCHI, S., GALLUS, G. & 4 others (1982). Oestrogens and obesity as risk factors for endometrial cancer in Italy. *Int. J. Epidemiol.,* **11,** 120.

LUBIN, F., RUDER, A.M., WAX, Y. & MODAN, B. (1985). Overweight and changes in weight throughout adult life in breast cancer etiology. *Am. J. Epidemiol.,* **122,** 579.

MCPHERSON, K. & DRIFE, J.O. (1986). The pill and breast cancer: Why the uncertainty? *Br. Med. J.,* **2,** 709.

NISKER, J.A., HAMMOND, G.L., DAVIDSON, B.J. & 4 others (1980). Serum sex-hormone-binding globulin capacity and the percentage of free estradiol in postmenopausal women with and without endometrial carcinoma. *Am. J. Obstet. Gynecol.,* **138,** 637.

PIKE, M.C. & ROSS, R.K. (1984). Breast cancer. *Br. Med. Bull.*, **40**, 351.
SIITERI, P.K. (1978). Steroid hormones and endometrial cancer. *Cancer Res.*, **38**, 4360.
THOMAS, D.B. (1984). Do hormones cause breast cancer? *Cancer*, **53**, 595.
WILLETT, W.C., BROWNE, M.L., BAIN, C. & 6 others (1985). Relative weight and risk of breast cancer among premenopausal women. *Am. J. Epidemiol.*, **122**, 731.